# Shear Strength Behaviors of Aeolian Sand Solidified by Microbially Induced Calcite Precipitation and Basalt Fiber Reinforcement

**DOI:** 10.3390/ma16175857

**Published:** 2023-08-26

**Authors:** Gang Li, Jia Liu, Jinli Zhang, Yiran Yang, Shufeng Chen

**Affiliations:** 1 Shaanxi Key Laboratory of Safety and Durability of Concrete Structures, Xijing University, Xi’an 710123, China; t_bag945@126.com (G.L.); yryangxa@163.com (Y.Y.); chensf1127@163.com (S.C.); 2 School of Geological Engineering and Geomatics, Chang’an University, Xi’an 710054, China; 3 State Key Laboratory of Coastal and Offshore Engineering, Dalian University of Technology, Dalian 116024, China; jlzhang@dlut.edu.cn

**Keywords:** aeolian sand, MICP, basalt fiber, shear strength, peak strength

## Abstract

Aeolian sand flow is identified as the main factor in the formation of sandstorms. However, conventional sand fixation methods cannot meet the current development requirements of environmental protection. In this paper, the method using Microbially Induced Calcite Precipitation (MICP) combined with basalt fiber reinforcement (BFR) was adopted to solidify the aeolian sand. Consolidated undrained triaxial shear tests were carried out to analyze the influence of fiber content, fiber length, confining pressure, and other factors on stress–strain characteristics, peak strength, brittleness index, and shear strength of aeolian sand. A shear strength model of aeolian sand solidification using MICP-BFR and considering the effect of fiber length and fiber content is established according to the test results. The results show that the peak strength of aeolian sand solidified by MICP-BFR is remarkably higher than that of aeolian sand solidified by MICP alone, and the peak strength rises with the increasing fiber length, fiber content, and confining pressure. The application of fiber can effectively reduce the brittleness index of aeolian sand solidified by MICP and improve the sample ductility. As fiber content and fiber length increase, the cohesion of solidified aeolian sand increases while the internal friction angle changes relatively little. In the limited range set by the test, the fiber length of 12 mm and the fiber content of 1.0% constitute the optimum reinforcement condition. The test results coincide with the model prediction results, indicating that the new model is fitting for predicting the shear strength of aeolian sand solidified by MICP-BFR. The research results provide an important reference value for guiding the practice of wind prevention and sand fixation in desert areas.

## 1. Introduction

Sandstorm is the primary cause of atmospheric pollution, surface vegetation destruction, and land desertification, resulting in a significant adverse impact on human life and economic development. Aeolian sand is formed as sandy soil due to aeolian deposits in the extremely arid and semi-arid environment of desert areas. Aeolian sand mobility is the primary origin of land desertification. Under the combined effect of strong wind and local thermal instability, sandstorm breaks out easily [1,2,3,4]. At present, the main sandstorm prevention methods contain engineering fixation methods, biological fixation methods, and chemical fixation methods. Engineering sand fixation methods have the disadvantage of burial by shifting sand and are often used for temporary and auxiliary sand fixations. Biological sand fixation methods suffer greater difficulty for longer periods. It is difficult to implement such methods in some arid and water-deficient areas. Chemical sand fixation methods have poor compatibility with desert environments and easily cause secondary pollution [5,6,7]. MICP technology has been an emerging method in recent years. It cements loose soil particles into a whole using the microbially induced calcium carbonate crystals, thus improving the soil’s mechanical behavior [8,9,10]. MICP fixation method can remarkably enhance the strength and stiffness of soil. However, obvious brittle failures are found in the solidified soil, and the soil durability is thus affected. However, the toughening mechanism of the fiber reinforcement method can significantly reduce the brittle failure of soil [11,12,13,14]. Based on specific studies, Zheng et al. [15] concluded that CaCO_3_ crystals generated by MICP deposits increased the roughness of the fiber surface; the mixture of CaCO_3_ and sand anchored the fibers, and the fibers reduced brittle failures of the soil solidified by MICP. With the increasing fiber content, both the strength and toughness of the sample show the change law of first increasing and then decreasing. The optimum fiber content is 0.3% to 0.5%. Qiu et al. [16] pointed out that the unconfined compressive strength of the solidified sand increases first and then decreases with the increasing content of carbon fiber, and the optimum fiber content for quartz sand and calcareous sand is 0.2% and 0.1%, respectively. Imran et al. [17,18] discovered that jute fiber could enhance the durability of the sand solidified through MICP by more than 50%, while dry–wet cycles adversely affect the properties of solidified sand. The application of jute fiber improves the ductility and toughness of the sand solidified by MICP, and fiber content produces a more significant effect than fiber length. According to the conclusion of Wang et al. [19], the tensile strength of the sand solidified by MICP shows the change law of first increasing and then decreasing with the increasing fiber content and fiber length. When the fiber content is 0.6%, the tensile strength of the solidified calcareous sand increases by about 172.4%, and the peak deformation improves by 158.1%. Based on specific studies, Tang et al. [20] discovered that the application of fibers has increased the unconfined compressive strength of the calcareous sand, brittleness, and ductility by 2.6, 3.5, and 5.0 times, respectively. When the fiber content reaches 0.4%, the residual strength of the sample is approximately 63.1% of the unconfined compressive strength. Choi et al. [21,22] pointed out that both the splitting tensile strength and secant modulus of elasticity of the solidified sand rise with the increasing of CaCO_3_ content or fiber ratio, and the application of fibers can remarkably enhance the failure strain of the sand solidified by MICP and the splitting tensile strength after failure. Fiber promoted the MICP process by bridging the pores between sand particles, and the brittleness was deduced by nearly half compared with that of untreated sand. According to the conclusion of Xiao et al. [23], both the unconfined compressive strength and splitting tensile strength of the sand solidified by MICP rise significantly with the increasing CaCO_3_ content after an appropriate amount of basalt fiber is added. However, the axial strain corresponding to peak failure deduces with the increasing CaCO_3_ content. Zhao et al. [24] reported that the axial strain of the sand solidified by MICP rises with the increasing fiber content but falls with the increasing CaCO_3_ content under peak failures. Yin et al. [25] discovered that the mechanical properties of MICP solidified sand could be improved significantly, and fibers could increase the colonization area of bacteria and the yield of CaCO_3_, improve the sample ductility and toughness, and reduce the stiffness. Liu et al. [26] concluded that the soil body solidified by MICP has weak ductility under the condition of dry–wet and freeze–thaw cycles and acid rain. The application of fibers has enhanced the ductility of the sample solidified by MICP. After five dry–wet cycles, the failure strain enhanced with fibers reaching 1.6%, while the failure rate of the unsolidified sample was only 0.4%. Zhao et al. [27] compared the effects of carbon fiber, basalt fiber, polypropylene fiber, and polyester fiber on the penetration and strength characteristics of soil solidified by MICP, summarized the positive effect of fibers on the generation of CaCO_3_ crystals and concluded that the four types of fiber would not change the type of CaCO_3_ crystals. In consideration of the effects of penetration, strength, and economic efficiency, the combination of basalt fiber and MICP is recommended for soil solidification. In light of the conclusion of Li et al. [28], both the unconfined compressive strength and shear strength of the solidified soil rise with the increasing fiber content, and the optimum fiber content is 0.2% to 0.3%. With the optimum fiber content, the unconfined compressive strength of the sample has been enhanced more than twice, and the failure strain has been increased by three times. Based on specific studies, Zhao et al. [29] discovered that after the application of glass fiber, polyester fiber, and jute fiber, the water absorption of the calcareous sand solidified by MICP falls by 11.60%, 21.18%, and 7.29%, respectively while the unconfined compressive strength rises by 24.20%, 60.76%, and 6.40% respectively. Polyester fiber is, therefore, recommended for the modification of calcareous sand. As proven by the relevant studies, Cheng et al. [30] discovered that after the addition of fibers, the unconfined compressive strength of the purple soil solidified by MICP rose significantly, and the soil toughness during failure was enhanced. The strength of the solidified soil shows the change law of first increasing and then decreasing with the increasing fiber content, and the optimum fiber content is 0.6%. Based on specific studies, Lv et al. [31] discovered that with the application of fibers, the sand solidified by MICP experiences the transformation from brittle failures to ductile failures; basalt fiber is the most fiber in improving the unconfined compressive strength, and polyester fiber results in a higher residual strength. Jiang et al. [32] pointed out that carbon fiber could significantly improve the strength of MICP-solidified calcareous sand and the yield of CaCO_3_ and increase the sample strength by 135% to 217% and the yield of CaCO_3_ by 15% to 34%. According to the conclusion of Li et al. [33], fibers can remarkably enhance the mechanical property and rain erosion resistance of the sand solidified by MICP; the peak penetration resistance shows the change law of first increasing and then decreasing with the increasing the fiber content, and the optimum fiber content is 0.2%. Yao et al. [34] concluded that wool fibers provide nucleation sites for the deposition of CaCO_3_, reduce the soil porosity of the sample, and increase the mechanical properties. When the fiber content is 0.1%, the compressive strength of the sample rises by 3.34 times, and the bending resistance also rises significantly. Spencer et al. [35] concluded that after an amount of jute fiber equal to 0.75% is added, the unconfined compressive strength rises by about six times, and the yield of CaCO_3_ rises by nearly three times. According to the study of Fang et al. [36], the dry density of the coral sand solidified by MICP increases between 1.38 g/cm^3^ and 1.70–2.01 g/cm^3^; the permeability decreases by two to three magnitude, and the unconfined compressive strength rises to 2.78–21.65 MPa after the application of fibers. As a result, the optimum fiber content is 0.2%, and the optimum fiber length is 9 mm or 12 mm. Based on specific studies, Lei et al. [37] reported that the unconfined compressive strength, tensile strength, and CaCO_3_ yield of the sand solidified by MICP rise consequently as increasing of fiber content, and carbon fibers are more effective than basalt fibers and glass fibers in sand solidification. Dikshit et al. [38] concluded that the compressive strength of lunar soil was significantly increased by treatment with MICP and guar gum, while the strength decreased a little with the addition of glass fiber. Espinal et al. [39] revealed that the fiber surface energy, as calculated by the water contact angle, was the main factor for bond strength. Chen et al. [40] reported that high fiber content can obtain a higher strength of MICP-treated peat soil, while high initial cementation resources can decrease the strength.

Basalt fiber has the characteristics of corrosion resistance, thermal resistance, environmental friendliness, low cost, high strength, and durability and is widely used in the fields of aerospace, manufacturing, and civil engineering. As demonstrated by the existing study results, the combination of MICP and fiber can remarkably enhance the strength, stiffness, toughness, and erosion resistance of the solidified soil. Most of the relevant studies focus on standard sand and calcareous standard. There are fewer studies combining aeolian sand with static characteristics. This paper primarily validates the feasibility of the MICP-BFR method to solidify the aeolian sand and establishes a shear strength model of aeolian sand considering fiber effects. By conducting the triaxial consolidated undrained triaxial shear test, this paper analyzes the effect of initial dry density (*ρ*_d_), fiber length (*FL*), fiber content (*FC*), confining pressure (*σ*_3_), and other factors on the stress–strain characteristics, peak strength, brittleness index, and shear strength of aeolian sand solidification and obtains the optimum reinforcement conditions of aeolian sand solidification through MICP. A shear strength model of aeolian sand solidification using MICP-BFR and considering the effect of fiber length and fiber content is established according to the results of the triaxial test. The research results can provide an important reference value for guiding the practice of wind prevention and sand fixation in desert areas.

## 2. Materials and Methods

### 2.1. Test Materials

**Figure 1:**
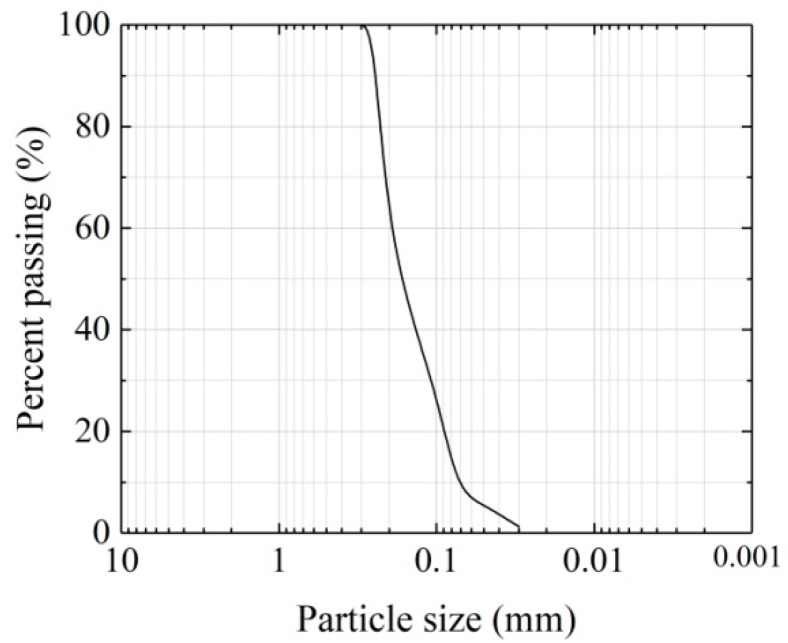
Grading curve of aeolian sand.

The aeolian sand for the test was gathered from the Ulanbh Desert of the Inner Mongolia Autonomous Region. Featuring a typical temperate continental climate, the desert area has low-frequency precipitation. The main physical properties of aeolian sand are shown in Table 1. Figure 1 shows the grading curve of aeolian sand; the aeolian sand is identified as poorly graded fine sand according to coefficient of nonuniformity and coefficient of curvature.

**Figure 2:**
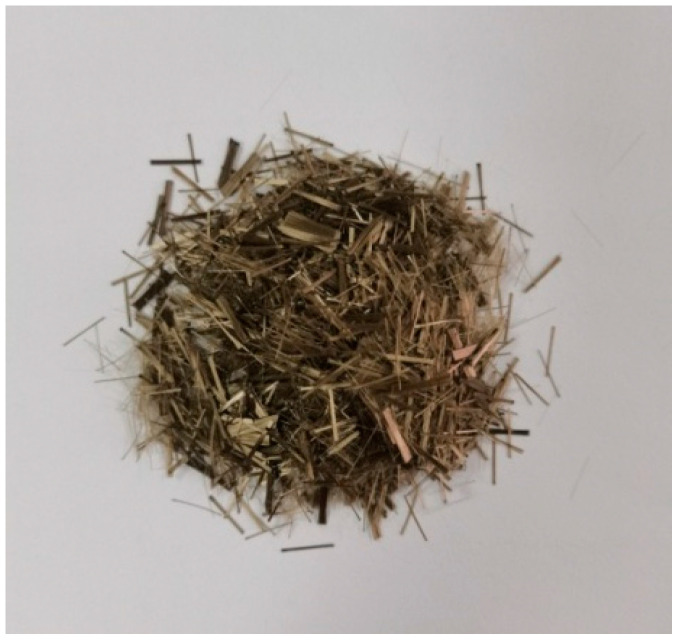
Basalt fiber.

Basalt fiber was bought from Shijiazhuang Zhuzhong Technology Co., Ltd. (Hebei, China), as shown in Figure 2. It is a new type of high-strength inorganic composite material resistant to high temperature and corrosion. It is characterized by high economic efficiency, environmental friendliness, and durability. Basalt fiber surfaces are smooth and bundled. After being decomposed, the fiber is fluffy and can sink into water. For the basalt fiber, the density is 2.65 g/cm^3^, the diameter is 10 μm, the tensile strength is 3500 to 4500 MPa, the elasticity modulus is 100 GPa, and the elongation at break is 2.2%.

*Sporosarcina pasteurii* (ATCC 11859) was used for the test, and the bacterium culture medium consisted of (1) 15 g tryptone, (2) 5 g peptone, and (3) 5 g sodium chloride. After 900 mL of deionized water was mixed with each component, the pH value of the medium was modified to 9, and subsequently, high-temperature and high-pressure sterilization was carried out. After the medium was sterilized and the temperature was lowered to 60 °C, 100 mL of 20% urea sterilized by filtration was added. The bacteria were cultured for several hours at a shaking frequency of 170 rpm and a temperature of 35 °C, and the absorbance of the bacterial solution at a wavelength of 600 nm was measured by a UV-visible spectrophotometer to control OD_600_ at 1.5. The mixed solution of urea and calcium chloride with equal concentration (1 mol/L) and equal volume (500 mL) was prepared as the cementing solution.

### 2.2. Sample Preparation

**Figure 3:**
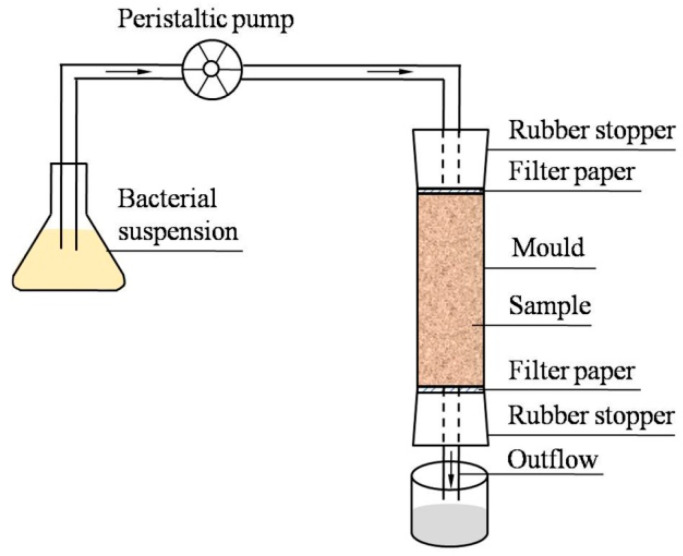
Schematic diagram of test device.

The organic glass tube with 39.1 mm in inner diameter and 150 mm in height was selected as the sample mold, which was cut in the middle to easily take out the solidified sample. In addition, the sample mold was tightened with a stainless steel clamp. The aeolian sand sample was 39.1 mm in diameter and 80 mm in height. During the test, a certain amount of aeolian sand was filtered through a 0.5 mm sieve and stored for further use. Then, the basalt fibers of specific length and content (weight percentage) were broken into filaments and immersed in a small amount of water for decomposition. Then, the basalt fiber was uniformly mixed with a fixed amount of aeolian sand. The mixture was added into the organic glass mold for 4 layers, and the mixture between layers was roughened. Before the sample was added, three layers of filter paper were tiled at the lower end of the sample. After the sample was added, one layer of filter paper was tiled at the upper end of the sample. Both the upper and lower ends of the mold are blocked with perforated rubber plugs. The preformed hole was connected with the fluid inlet and fluid outlet. In order to prevent the side wall from leakage during perfusion, a plastic sheet was placed between the sand sample and the mold. During the experiment, the bacterial and cementing solutions were perfused into the sample by a peristaltic pump at the rate of 2 mL/min. Figure 3 shows the schematic diagram of the perfusion device. In the test, the method of repeated perfusion at intervals was used. In detail, the bacterial solution was perfused 1 time and kept still for 3 h; the cementing solution was perfused 4 times at regular intervals of 16 h. After the perfusion was completed, the sample was flushed 3 times with deionized water to terminate the MICP reaction and eliminate mineralization products.

### 2.3. Test Methodology

After being fully solidified, the sample was naturally dried indoors for 10 days. Subsequently, a triaxial consolidated undrained (CU) test was performed on the aeolian sand sample in accordance with the Standard for geotechnical testing method (GB/T 50123-2019) [41]. During the test, the sample was first consolidated and then loaded at the strain rate of 0.5% per minute. The test was terminated when the axial strain reached 15%. The test plan is shown in Table 2.

## 3. Results and Discussion

### 3.1. Stress–Strain Characteristic Analysis of Aeolian Sand

**Figure 4:**
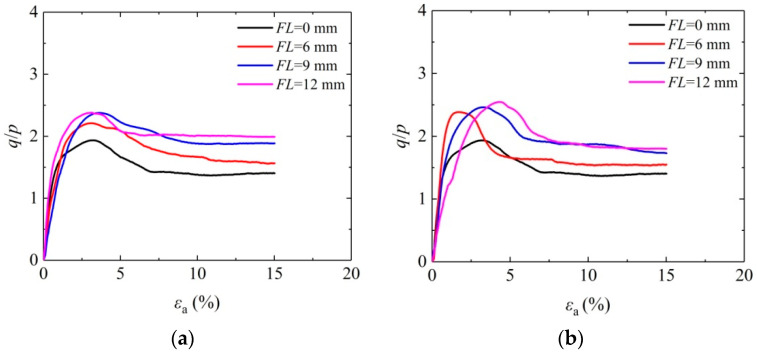
Effect of fiber length on the stress–strain curve of solidified aeolian sand: (**a**) *FC* = 0.6%; and (**b**) *FC* = 1.0%.

Figure 4 shows the curve of effect caused by fiber length on the average stress ratio (*q*/*p*) and axial strain of solidified aeolian sand when the confining pressure is 100 kPa so that the effect of adding fibers on the mechanical properties of solidified aeolian sand is analyzed. Actually, *p* is the average principal stress, and *q* is the generalized shear stress. It can be observed from the figure that the stress–strain curve of solidified aeolian sand shows the characteristics of strain softening, and the average stress ratio of the solidified aeolian sand has been improved significantly. With the steady rise of axial strain, the average effective stress ratio reaches the peak and then falls gradually. The peak stress ratio of the sand solidified by MICP-BFR obviously lags behind that of the unreinforced sample, indicating that the application of fibers has remarkably improved the sample toughness. The peak average stress ratio of the aeolian sand solidified by MICP-BFR rises with increasing fiber length. When the fiber length is 12 mm, the aeolian sand has the maximum peak average stress ratio. The above situation arises from the “bridging” role [34] of fibers in the sample. Fibers are anchored between sand particles by CaCO_3_ crystals, thus ensuring an excellent overall mechanical behavior for the solidified aeolian sand.

**Figure 5:**
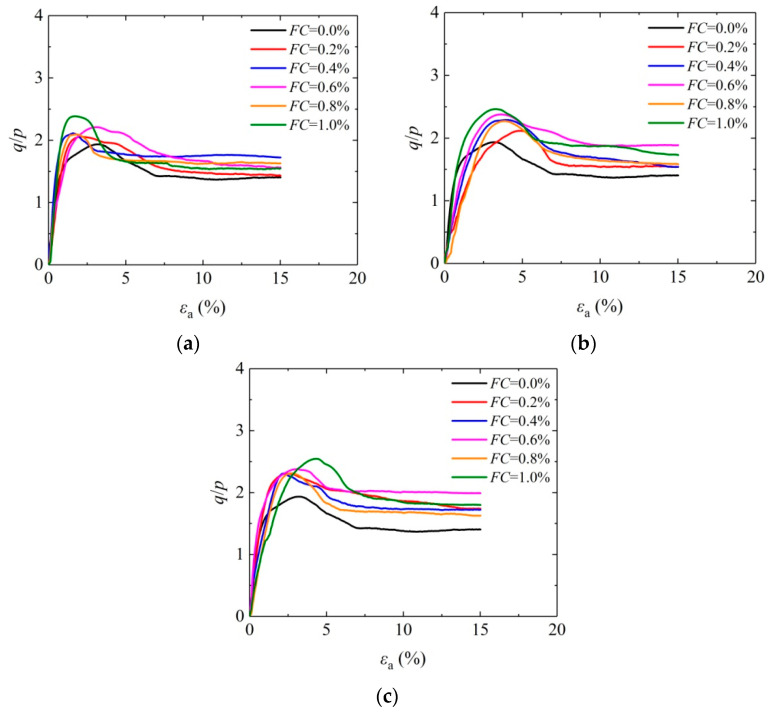
Effect of fiber content on the stress–strain curve of solidified aeolian sand: (**a**) *FL* = 6 mm; (**b**) *FL* = 9 mm; and (**c**) *FL* = 12 mm.

Figure 5 shows the stress–strain effect curve of fiber content on the solidified aeolian sand when the confining pressure is 100 kPa, and the fibers have different lengths (6 mm, 9 mm, and 12 mm). It can be seen from the figure that the peak average stress ratio of the solidified aeolian rises with the increasing fiber content. When the fiber content is 1.0%, the aeolian sand has the maximum peak average stress ratio. As for the primary cause of the above situation, the interwoven spatial mesh structure has restricted the deformation of sand particles under the cementing effect of CaCO_3_ crystals with the increasing fiber content and fiber amount when the fiber length is kept constant, thereby improving the strength of solidified aeolian sand.

**Figure 6:**
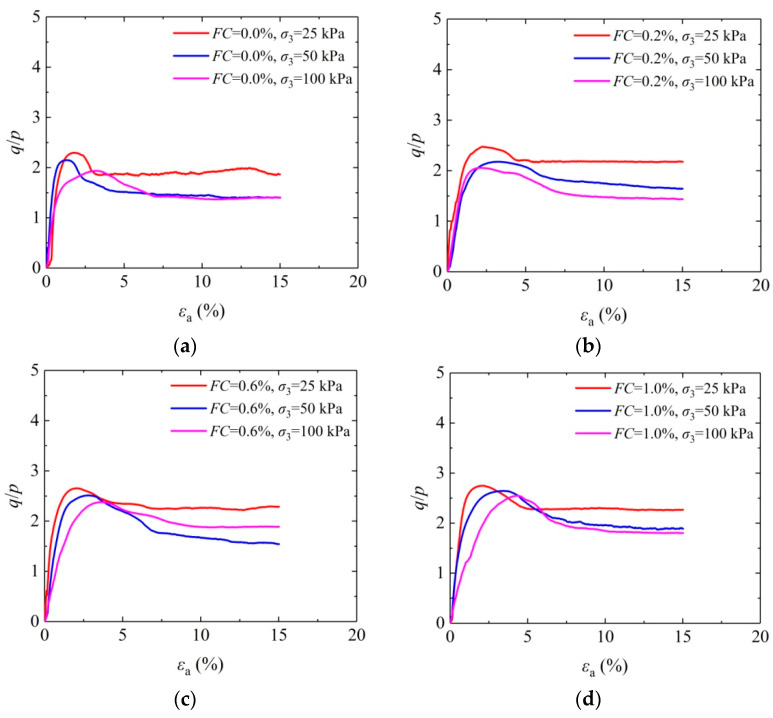
Effect of confining pressure on the stress–strain curve of solidified aeolian sand: (**a**) *FL* = 0 mm; (**b**) *FL* = 6 mm; (**c**) *FL* = 9 mm; and (**d**) *FL* = 12 mm.

With the increasing in situ depth of soil, the lateral pressure rises. In this view, confining pressure has a significant effect on soil strength. Figure 6 shows the effect of confining pressure on the stress–strain curve of the solidified aeolian sand when the fiber length is 0, 6, 9, and 12 mm and the fiber content is 0.0%, 0.6%, and 1.0%. As shown in the figure, for the aeolian sand solidified by MICP and MICP-BFR, the peak average stress ratio is apt to decrease gradually as the increasing confining pressure and the corresponding axial strain shows an overall increasing trend, showing that the resist deformation ability of the sample has been improved to some extent under higher confining pressure. With regard to the primary cause, the contact between fiber and sand particles becomes closer, and more contact points appear with an increasing confining pressure. Under the cementing effect of CaCO_3_ crystals, the cementing strength and friction strength become higher. When the sample is under shear action, the spatial mesh structure interwoven with fibers constitutes a broader force transfer system. Both force conversion effect and force transfer effect occur between fibers and sand particles. Then, fibers and sand particles jointly bear external loads, thus enhancing the strength of aeolian sand [42].

### 3.2. Peak Strength Analysis of Aeolian Sand

Table 3 shows the statistics regarding the peak strength of solidified aeolian sand under different fiber lengths, fiber contents, and confining pressures. As can be observed from the table, the peak strength of the solidified aeolian sand sample rises steadily as the increasing fiber length, fiber content, and confining pressure. Based on comparisons, it is discovered that the peak strength of the aeolian sand solidified by MICP-BFR has been improved significantly. At 25 kPa confining pressure, the peak strength of the aeolian sand solidified by MICP is 244.88 kPa. When the fiber length is 6 mm, and the fiber content is 0.2%, the peak strength of the solidified aeolian sand is 352.88 kPa. When the fiber length is 12 mm and the fiber content is 1.0%, the peak strength of the aeolian sand reaches 815.87 kPa and increases by 2.33 times compared with unreinforced samples. Zheng et al. [43] studied the peak strength of the standard microbially solidified and reinforced with basalt fibers sand and discovered that the application of fibers could significantly improve the peak strength of samples, which coincides with the conclusion in this paper. However, the rules of peak strength variation with fiber length and fiber content are different. The peak strength shows the change trend of first increasing and then decreasing with the increasing fiber length and fiber content. This situation may arise from the greater difference in physical properties of the sand selected for this test, and sand particle diameter and sample porosity have a greater effect on the MICP solidification [43].

### 3.3. Brittleness Index Analysis of Aeolian Sand

To analyze whether the application of fibers can significantly reduce the brittle failure of aeolian sand treated by MICP, the brittleness index is used to measure the effect of fiber length and fiber content on the ductility of solidified aeolian sand. As the index decreases, particularly approaching zero, the failure behavior becomes increasingly ductile. Brittle index *I_B_* [44] is expressed as follows:

(1) $$I_{B}=\frac{{\left(q/p\right)}_{f}}{{\left(q/p\right)}_{u}}-1$$

where *I_B_* is the brittleness index; (*q*/*p*)_f_ is the failure stress ratio, and (*q*/*p*)_u_ is the ultimate stress ratio.

**Figure 7:**
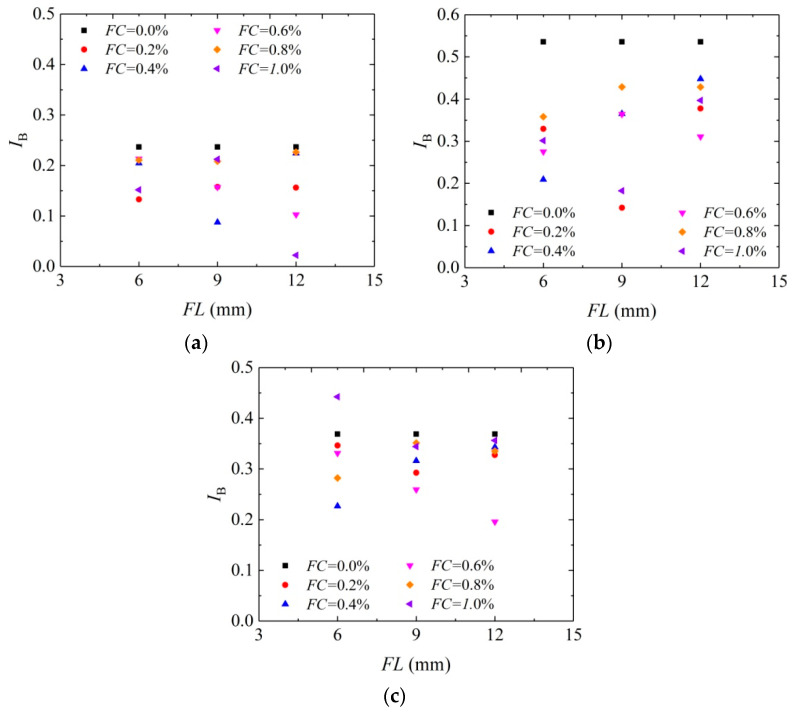
Curve of relation between brittleness index and fiber length: (**a**) *σ*_3_ = 25 kPa; (**b**) *σ*_3_ = 50 kPa; and (**c**) *σ*_3_ = 100 kPa.

Figure 7 shows the curve of relations between the brittleness index of solidified aeolian sand and fiber length under the confining pressures of 25 kPa, 50 kPa, and 100 kPa. In conclusion, the application of fiber can significantly decrease the brittleness index of aeolian sand solidified by MICP, indicating that fibers help improve the sample ductility. It coincides with the results of Liu et al. [45]. When the fiber length is 9 mm and 12 mm, the samples with a larger amount of fibers have a lower brittleness index under different confining pressures. For the aeolian sand sample under 25 kPa confining pressure, fiber length of 12 mm, and fiber content of 1.0%, the peak strength is 815.87 kPa, and the brittleness index is only 0.02. In comparison with the brittleness index of the sample solidified by MICP, the brittleness index has declined significantly. For the aeolian sand sample under 50 kPa confining pressure, fiber length of 9 mm, and fiber content of 1.0%, the peak strength reaches 955.50 kPa, and the brittleness index is 0.18. In comparison with the brittleness index of the sample solidified by MICP, the brittleness index has declined by 67%. According to the combined analysis of peak strength and brittleness index, the aeolian sand sample solidified by MICP-BFR with large peak strength has a lower brittleness index. This situation is more significant when the confining pressure is lower.

### 3.4. Shear Strength Index Analysis of Aeolian Sand

Based on the Mohr–Coulomb criterion, the cohesion and internal friction angle are the intercept and inclination of the strength envelope, respectively. Table 4 sums up the shear strength (overall strength) index of the solidified aeolian sand under different fiber lengths and fiber contents. It can be observed from the table that the shear strength of the aeolian sand solidified by MICP-BFR is increased significantly compared with that of the aeolian sand treated by MICP. It indicates that the application of fibers has improved the solidification effect of MICP [33]. As increasing fiber content and fiber length, the cohesion of solidified aeolian sand shows a trend of steady increase and reaches the maximum when *FL* is 12 mm and *FC* is 1.0%, while the variation of internal friction angle is significantly small. As for the primary cause of the above situation, the CaCO_3_ crystals generated by MICP have filled the pores and cemented the loose sand particles into a whole, thus improving the cohesion. In addition, the CaCO_3_ material on the sand particle surface has further roughened and improved the friction force. With the application of fibers, there are more contact points between longer fibers and sand particles. Then, the force conversion effect and the force transfer effect occur between sand particles and fibers to constitute a broader force transfer system and ensure a better overall mechanical behavior.

### 3.5. Shear Strength Modeling of Aeolian Sand

(a) Modeling

According to the analysis of Table 4, the cohesion of the aeolian sand solidified by MICP-BFR is larger than that of the sample solidified by MICP. For aeolian sand solidified by MICP-BFR, it can be concluded that the cohesion significantly increased with an increase in fiber content and fiber length, while the internal friction angle changed little. It is assumed that there is a correlation of cohesion between the aeolian sand solidified by MICP-BFR and the aeolian sand solidified by MICP, and the cohesion is the function that the product of fiber length and fiber content is divided by fiber diameter, which is expressed as follows:

(2) $$c_{F}=f\left(\frac{FL\cdot FC}{d}\right)c_{0}$$

where *c*_F_ is the cohesion of the aeolian sand treated by MICP-BFR (kPa); *c*_0_ is the cohesion of the aeolian sand treated by MICP (kPa); *FL* is fiber length (mm); *FC* is fiber content (%), and *d* is fiber diameter (mm).

The transformation of Equation (2) yields:

(3) $$\frac{c_{F}}{c_{0}}=f\left(\frac{FL\cdot FC}{d}\right)$$

Based on data analysis, it has been discovered that *c*_F_/*c*_0_ increases linearly with the increasing *FL*·*FC*/*d*. Equation (3) can be expressed as follows:

(4) $$\frac{c_{F}}{c_{0}}=\alpha \frac{FL\cdot FC}{d}+\beta$$

where *α* and *β* are the model parameters that can be obtained by regression analysis of test data.

The reorganization of Equation (4) yields

(5) $$c_{F}=\left(\alpha \frac{FL\cdot FC}{d}+\beta \right)c_{0}$$

Compared with the aeolian sand solidified by MICP, the internal friction angle of aeolian sand solidified by MICP-BFR changes less significantly as increasing fiber length and fiber content, thereby concluding that

(6) $$\tan\varphi_{F}=\tan\varphi_{0}$$

where *φ*_F_ is the internal friction angle of the aeolian sand solidified by MICP-BFR (°), and *φ*_0_ is the internal friction angle of the aeolian sand solidified by MICP (°).

According to the Mohr–Coulomb strength criterion, the shear strength of the aeolian sand sample solidified by MICP-BFR is calculated as follows:

(7) $$\tau_{F}=\sigma \tan\varphi_{F}+c_{F}$$

where *τ_F_* is the shear strength of the aeolian sand treated by MICP-BFR (kPa), and *σ* is the principal stress (kPa).

The shear strength model of the aeolian sand solidified by MICP-BFR is deduced by putting Equations (5) and (6) into Equation (7):

(8) $$\tau_{F}=\sigma \tan\varphi_{0}+\left(\alpha \frac{FL\cdot FC}{d}+\beta \right)c_{0}$$

(b) Parameter acquisition

**Figure 8:**
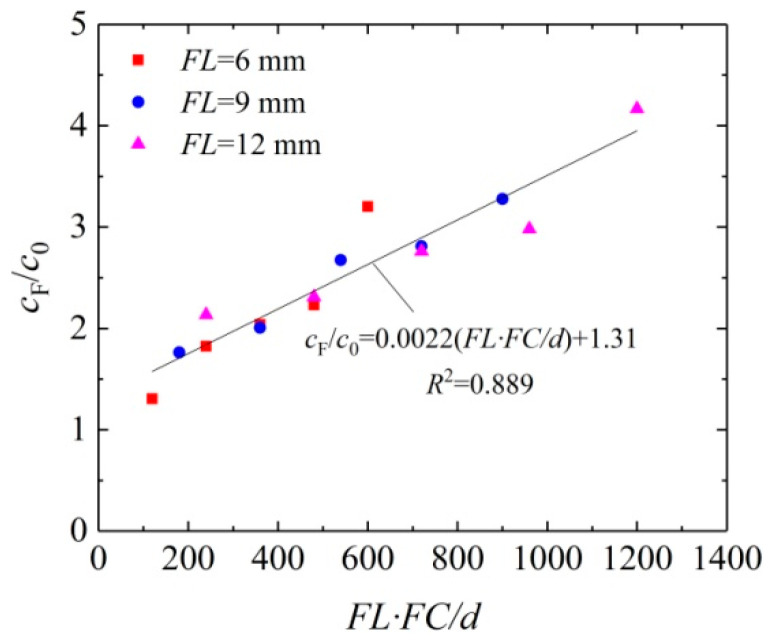
Curve of relation between *c*_F_/*c*_0_ and *FL*·*FC*/*d*.

Figure 8 shows the curve of relation between *c*_F_/*c*_0_ and *FL*·*FC*/*d*. As shown in the figure, *c*_F_/*c*_0_ rises with the increasing *FL*·*FC*/*d,* and a linear relation exists between them with a determination coefficient of 0.889, indicating a good correlation between *c*_F_/*c*_0_ and *FL*·*FC*/*d*. According to the figure, we can read that the value of parameter *α* and *β* is 0.0022 and 1.31, respectively.

(c) Model verification

**Figure 9:**
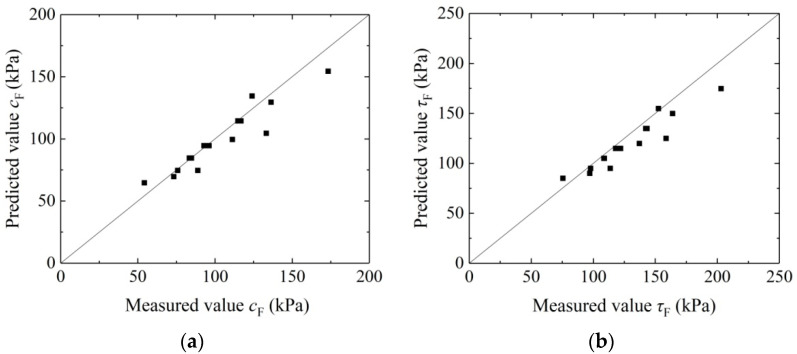
Comparison between model calculation results and test results: (**a**) cohesion; and (**b**) shear strength.

To validate the reliability of the above model, the known data of *c*_0_, *FL*, *FC,* and *d* are combined, and the parameters *α* and *β* are put into Equation (5) to gain the cohesion *c*_F_ of the aeolian sand solidified by MICP-BF. According to the known *φ*_0_ and Equation (8), we have calculated and obtained the shear strength of the aeolian sand solidified via MICP-BFR. Figure 9a shows the result comparison between the cohesion test and model calculation, and Figure 9b shows the result comparison between the shear strength test and model calculation. It can be observed that the model calculation results coincide with the experiment results, indicating that the model is fitting for predicting cohesion and shear strength of aeolian sand solidified by MICP-BFR.

## 4. Conclusions

With the aeolian sand samples respectively solidified by MICP and MICP-BFR, we have conducted a consolidated undrained triaxial shear test to investigate the effect of fiber content, fiber length, confining pressure, and other factors on stress–strain characteristics, peak strength, brittleness index, and shear strength of aeolian sand solidification, and finally worked out the optimum reinforcement condition for the aeolian sand solidification through MICP. A shear strength model of aeolian sand solidification using MICP-BFR and considering the effect of fiber length and fiber content is established according to the test results. The main conclusions are as follows:

(1) The stress–strain curve of the aeolian sand solidified by MICP and MICP-BFR shows the characteristics of strain softening. The peak strength of aeolian sand solidified by MICP-BFR is significantly higher than that of aeolian sand treated by MICP alone, and the peak strength solidified by MICP-BFR rises with the increasing fiber length, fiber content, and confining pressure. In the range set by the test, the fiber length of 12 mm and the fiber content of 1.0% constitute the optimum reinforcement condition;

(2) The application of fiber can significantly decrease the brittleness index of aeolian sand solidified by MICP and improve the sample ductility. The sample with a larger fiber length and higher fiber content has better ductility but a lower brittleness index;

(3) As fiber content and fiber length increase, the cohesion of solidified aeolian sand shows a trend of steady increase and reaches the peak when the fiber’s length is 12 mm, and the fiber content is 1.0%, while the variation of internal friction angle is little;

(4) The test results coincide with the model calculation results, validating the reliability of the new model and indicating that the model is fitting for predicting the shear strength of aeolian sand solidified by MICP-BFR;

(5) Furthermore, the in situ and aging tests will be conducted in a future study to further validate the feasibility of the MICP-BFR method to solidify the aeolian sand.

## Figures and Tables

**Table 1:** Physical properties of aeolian sand.

*G*_s_	*ρ*_dmax_ (g/cm^3^)	*ρ*_dmin_ (g/cm^3^)	*d*_10_ (mm)	*d*_30_ (mm)	*d*_60_ (mm)	*C*_u_	*C*_c_
2.65	1.85	1.47	0.073	0.109	0.187	2.58	0.88

**Table 2:** Scheme of the aeolian sand solidification test.

*σ*_3_ (kPa)	*ρ*_d_ (g/cm^3^)	Cementation Number	Fiber Length (mm)	Fiber Content (%)
25, 50, 100	1.6	12	0	0.0
			6	0.2, 0.4, 0.6, 0.8, 1.0
			9	0.2, 0.4, 0.6, 0.8, 1.0
			12	0.2, 0.4, 0.6, 0.8, 1.0

**Table 3:** Peak strength of solidified aeolian sand.

*ρ*_d_ (g/cm^3^)	*FL* (mm)	*FC* (%)	*σ*_3_ (kPa)	*q*_peak_ (kPa)	*ρ*_d_ (g/cm^3^)	*FL* (mm)	*FC* (%)	*σ*_3_ (kPa)	*q*_peak_ (kPa)
1.6	0	0	25	244.88	1.6	9	0.4	50	632.78
1.6	0	0	50	378.42	1.6	9	0.6	50	771.66
1.6	0	0	100	544.47	1.6	9	0.8	50	801.90
1.6	6	0.2	25	352.88	1.6	9	1	50	955.50
1.6	6	0.4	25	407.66	1.6	9	0.2	100	717.99
1.6	6	0.6	25	439.31	1.6	9	0.4	100	966.64
1.6	6	0.8	25	537.99	1.6	9	0.6	100	1143.91
1.6	6	1	25	768.08	1.6	9	0.8	100	1245.31
1.6	6	0.2	50	397.13	1.6	9	1	100	1371.56
1.6	6	0.4	50	559.23	1.6	12	0.2	25	479.07
1.6	6	0.6	50	617.57	1.6	12	0.4	25	514.14
1.6	6	0.8	50	778.40	1.6	12	0.6	25	621.37
1.6	6	1	50	948.27	1.6	12	0.8	25	719.14
1.6	6	0.2	100	653.56	1.6	12	1	25	815.87
1.6	6	0.4	100	716.04	1.6	12	0.2	50	717.95
1.6	6	0.6	100	839.50	1.6	12	0.4	50	737.18
1.6	6	0.8	100	923.63	1.6	12	0.6	50	810.20
1.6	6	1	100	1162.48	1.6	12	0.8	50	805.25
1.6	9	0.2	25	443.51	1.6	12	1	50	1109.99
1.6	9	0.4	25	498.83	1.6	12	0.2	100	997.53
1.6	9	0.6	25	570.62	1.6	12	0.4	100	1004.83
1.6	9	0.8	25	672.12	1.6	12	0.6	100	1150.24
1.6	9	1	25	798.11	1.6	12	0.8	100	1342.59
1.6	9	0.2	50	608.92	1.6	12	1	100	1682.75

**Table 4:** Shear strength index of aeolian sand sample.

*ρ*_d_ (g/cm^3^)	*FL* (mm)	*FC* (%)	*c* (kPa)	*φ* (^o^)
1.6	0	0	41.60	39.20
	6	0.2	54.28	40.44
	6	0.4	75.83	41.35
	6	0.6	84.82	43.96
	6	0.8	92.87	45.26
	6	1.0	133.22	45.51
	9	0.2	73.33	43.67
	9	0.4	83.41	45.24
	9	0.6	111.29	45.97
	9	0.8	116.86	46.64
	9	1.0	136.32	47.84
	12	0.2	88.84	44.86
	12	0.4	96.13	46.01
	12	0.6	114.86	47.71
	12	0.8	124.06	48.71
	12	1.0	173.38	49.88

## Data Availability

Not applicable.
